# A LC-MS/MS Assay for Quantification of Amodiaquine and Desethylamodiaquine in Dried Blood Spots on Filter Paper

**DOI:** 10.1155/ianc/5130424

**Published:** 2025-06-05

**Authors:** Natpapat Kaewkhao, Joel Tarning, Daniel Blessborn

**Affiliations:** ^1^Mahidol Oxford Tropical Medicine Research Unit, Faculty of Tropical Medicine, Mahidol University, Bangkok, Thailand; ^2^Centre for Tropical Medicine & Global Health, Nuffield Department of Clinical Medicine, University of Oxford, Oxford, UK

**Keywords:** amodiaquine, dried blood spots, LC-MS/MS, malaria, validation

## Abstract

Artesunate–amodiaquine (ARS–AQ) is a first-line antimalarial treatment recommended by the World Health Organization. AQ is the long acting partner drug in this combination, and therapeutic success is correlated with the terminal exposure to AQ. Dried blood spot (DBS) sampling for AQ is a convenient and minimally invasive technique, especially suitable for clinical studies in resource limited settings and pediatric studies. Our primary aim was to develop and validate a bioanalytical method for quantification of AQ and its active metabolite in capillary blood applied onto filter paper as a DBS sample. The separation was achieved using a reverse phase column (Zorbax SB-CN 50 × 4.6 mm, I.D. 3.5 μm) and a mobile phase consisting of acetonitrile:ammonium formate 20 mM with 0.5% formic acid (15:85, v/v). A 50 μL DBS was punctured with five 3.2 mm punches from the filter paper, and the punches collected correspond to approximately 15 μL of dried blood. The blood was then extracted using a mixture of 0.5% formic acid in water:acetonitrile (50:50, v/v), along with stable isotope-labeled internal standards (AQ-D10 and desethylamodiaquine [DAQ]-D5). Mass spectrometry was used for quantification over the range of 2.03–459 ng/mL for AQ and 3.13–1570 ng/mL for DAQ. The validation of the method was carried out in compliance with regulatory requirements. The intra- and interbatch precisions were below 15% and passed all validation acceptance criteria. No carryover and no matrix effects were detected. Normalized matrix factors (analyte/internal standard) ranged from 0.96 to 1.03 for all analytes, hence no matrix effects. AQ and DAQ were stable in all conditions evaluated. Long-term stability in DBS samples was demonstrated for up to 10 years when stored at −80°C and for 15 months when stored at room temperature. The developed method was demonstrated to be reliable and accurate. This assay may be particularly useful in the context of resource limited settings and in pediatric field studies.

## 1. Introduction

Malaria remains a significant global health challenge, resulting in an estimated 263 million cases and 597,000 deaths in 2023 [1]. Artesunate–amodiaquine (ARS–AQ) is a first-line treatment for acute uncomplicated *falciparum* malaria and also used in combination with sulfadoxine–pyrimethamine (SUD–PYM) for the prevention of malaria in endemic regions [2, 3]. ARS is a potent and fast-acting antimalarial, while AQ is less potent but long-acting partner drug. AQ has similar structure to chloroquine, with the only difference being a phenol ring in the molecular structure. This additional phenol ring leads to accumulation of AQ inside the *P. falciparum* parasite with up to 2-3 times higher accumulation than for chloroquine [4, 5]. In vivo, AQ rapidly converts to its primary active metabolite, desethylamodiaquine (DAQ), which confers most of the antimalarial activity. In the parasite's food vacuole, it can exist in both protonated (charged) and unprotonated (uncharged) forms. The charged form of AQ interacts with heme, a byproduct of hemoglobin digestion, inhibiting parasite heme polymerization and ultimately leading to parasite death [5–7]. Since AQ is concentrated in erythrocytes and binds to platelets and leucocytes, peak concentrations of AQ and DAQ are approximately 2-3 times higher in whole blood than in plasma [8]. AQ is eliminated relatively fast from the body resulting in a terminal elimination half-life of approximately 16 h, and its metabolite DAQ has a longer terminal elimination half-life of 12 days [9, 10]. Combining the quick acting but rapidly eliminated ARS with the prolonged exposure to AQ/DAQ leads to fast initial parasite reduction and sustained eradication of residual parasites to prevent recrudescence. Prolonged duration and exposure to AQ/DAQ and its relationship to therapeutic success supports the need for sensitive and accurate drug measurements in special populations [9, 10].

Two studies used conventional UV detection with limited sensitivity to measure the amount of AQ in plasma samples [11–13]. However, it is important to measure AQ and DAQ at lower concentrations for therapeutic monitoring of treatment regimens. For this reason, mass spectrometry would be better suited to achieve drug quantification at low concentrations [14]. Plasma have traditionally been the sample matrix of choice but it often requires a larger volume of blood of 200–500 μL to be collected, which is not always feasible in vulnerable populations like small children. Plasma and whole blood also require a continuous cold chain from sample collection to analysis, which is difficult in resource-limited settings [15, 16]. The advantages of small volume capillary blood sample collection, stored as dried blood spots (DBSs), include a feasible and simple sample collection and easier storage and transportation, and it also minimize the risk of infection of various pathogens for healthcare workers and laboratory staff [17, 18]. The small volume of blood acquired through heel or finger prick make it ideally suitable for newborns/children in clinical studies [17, 18]. However, the use of DBS technique together with liquid chromatography-tandem mass spectrometry (LC-MS/MS) also comes with some challenges, e.g., the small sample volume often leads to lower sensitivity [19, 20], and variable hematocrit levels, nonhomogenous blood spot, and the type of filter paper used can affect the results. These are the main causes of nonconsistency in the preanalysis steps and can impact the reliability of DBS drug quantification. However, the DBS technique is widely studied and its use is likely to increase in the future [21]. Using clear and well written sample handling procedures can minimize the preanalytical variations. A few published methods used DBS and UV or LC-MS/MS. However, these methods required a blood volume of 100–200 μL on filter paper, which is not always possible in pediatric patients [22, 23]. Using a fast and direct extraction method may not give sufficiently clean samples [19]. The traditional method of sample preparation is protein precipitation, but it is not applicable to DBS and the ability to remove phospholipids or other interferences through direct protein precipitation may not be adequate for MS/MS detection. Interfering residues can be resolved with techniques like solid phase extraction or liquid–liquid extraction, but these methods are time-consuming and labor-intensive. The aim of this study was to develop a simple extraction technique from a DBS of approximately 50 μL, followed by a quick and simplified sample clean up to eliminate interferences when using LC-MS/MS quantification. This would offer practical improvements to existing analytical assays, allowing better sensitivity, automation, faster analysis, and a reduced sample volume, particularly benefiting underserved populations such as young children and pregnant women.

## 2. Materials and Methods

### 2.1. Ethical Conduct of Research

Ethical approval for collecting blank human volunteer blood in this study was granted from the ethics committee of the Faculty of Tropical Medicine, Mahidol University, Bangkok, Thailand (approval number MUTM 2020-068-04). Data collection started on April 2024. Informed consent was obtained in writing. Blood donors who showed interest in participating in the study signed written consent forms and underwent screening assessments, including vital sign measurements and routine laboratory blood tests. The donated blood tubes were blinded at the Healthy Volunteer Ward and then transferred to the clinical laboratory.

### 2.2. Chemicals and Reagents

All reference standards, AQ (purity 100%), DAQ (purity 99.6%) and stable isotope-labeled internal standards (SIL) (AQ-D10 and DAQ-D5 purity > 99%) were acquired from AlsaChim (Illkirch, France). Purified water was produced using the Direct-Q 5 UV water system (Ultrapure water; UPW18, 2 MΩ·cm; Merck, Germany). MS grade acetonitrile and methanol, formic acid (98%–100%), and ammonium formate were obtained from Honeywell (formerly Fluka) (Charlotte, NC, USA).

### 2.3. Preparation of Standards, Working Solutions, Calibration Standards, and Quality Control DBS Samples

Stock solutions: 1 mg/mL (base form) of AQ, DAQ, AQ-D10 and DAQ-D5 were prepared in acetonitrile:water (50:50, v/v) containing 0.5% formic acid and stored at −80°C. Working solutions were diluted in acetonitrile:water (50:50, v/v) and used for spiking of whole blood. All solutions were allowed to equilibrate to room temperature (RT) before use.

The DBS calibration curve range of AQ and DAQ were set to 2.03–459 and 3.13–1570 ng/mL, respectively. Three quality control (QC) levels of AQ (6.12, 184, and 383 ng/mL) and DAQ (9.75, 603, and 1312 ng/mL) were prepared as DBS. In addition, DBS samples were prepared for the lowest and highest concentrations in the calibration range representing the lower limit of quantification (LLOQ) and upper limit of quantification (ULOQ). For dilution integrity, overcurve samples were prepared at about 2-3 × ULOQ and diluted five times with a blank DBS before analyzed (one spot punch of overcurve sample with four spots punch of blank DBS). The final volume of solvent in blank blood was kept below 5% in all samples. Prepared spiked blood were spotted as 50 μL blood spots on chromatography filter paper 31 ET Chr (Whatman, Buckinghamshire, UK) and allowed to dry completely for approximately 1-2 h at RT (∼50% RH, 22°C). The drying process was confirmed by visual inspection, ensuring a uniform appearance without wetness or glossiness. The blood appeared darker and duller compared with its freshly spotted state. Once dried, the blood spots were stored at ambient temperature and in −80°C until the day of analysis. Whole blood with EDTA as anticoagulant was used throughout the validation. Alternative filter paper Whatman DMPK-C, 903 Protein saver, and 3 MM Chr (Whatman, Buckinghamshire, UK) were also evaluated.

### 2.4. Extraction Procedure

An automated liquid handler platform (Freedom Evo 100, TECAN, Mannedorf, Switzerland) was used for the sample preparation process. A Robotic Punch Instrument (BSD600-Duet Semi Automated, Queensland, Australia) was used to punch out five discs of 3.2 mm in diameter from a single DBS (equivalent to about 15 μL of whole blood) into a 96-well plate. Then, 200 μL of extraction solution (0.5% formic acid in water:acetonitrile (50:50, v/v) containing stable isotope-labeled internal standards (AQ-D10 and DAQ-D53.44 ng/mL) was added. The sample plate was mixed on a Mixmate (Eppendorf, Hamburg, Germany) (1000 rpm, 10 min) and centrifuged (1100 ×g, 2 min). Acetonitrile (200 μL) was added to each sample and the plate was mixed once more on a Mixmate (1000 rpm, 2 min) followed by centrifugation (1100 ×g, 2 min). The extracted samples (250 μL) were loaded on a Phree Phospholipids Removal 96-well plate (8ES133-TGB, Phenomenex, CA, USA). Vacuum was applied until the entire sample volume had passed through the column, and the collected eluate was then diluted with 170 μL of water [24].

### 2.5. LC-MS/MS

A Dionex Ultimate 3000 system consisting of a binary LC pump, a vacuum degasser (LPG-3400RS), a temperature-controlled microwell plate autosampler set at 10°C (WSP-3000TBRS) and a temperature-controlled column compartment set at 40°C (TCC-3000RS) (Thermo Scientific, MA, USA). Data acquisition and processing were performed using Analyst 1.7 (Sciex, MA, USA). The analytes were separated on a Zorbax SB-CN 50 mm × 4.6 mm, I.D. 3.5 μm (Agilent Technologies), with a precolumn CN AJO-4305 4 mm × 3 mm, I.D. 3.5 μm (Phenomenex, Torrance, California, USA), at a flow rate of 700 μL/min. The compounds were separated and analyzed under isocratic conditions using 100% of mobile phase A (acetonitrile:ammonium formate 20 mM with 0.5% formic acid, 15:85, v/v), for 2.2 min (0–2.2 min), followed by immediate switch to a washout using 100% of mobile phase B, methanol: acetonitrile (75:25, v/v), for 1.7 min (2.2–3.9 min), after which the mobile phase was immediately returned to 100% A and allowed to equilibrate (3.9–6.5 min) until the next sample injection. The total runtime was 6.5 min per sample and the injection volume was 2 μL [24].

An API 5000 triple quadrupole mass spectrometer (Sciex, MA, USA) with a TurboV ionization source interface, operated in the positive ion mode, was used for the MS/MS analysis. Ion spray voltage was set to 5500 V, with a drying temperature at 650°C. The curtain gas (CUR) was 25 psi and the nebulizer (GS1) and auxiliary (GS2) gases 60 psi [24].

**Figure 1:**
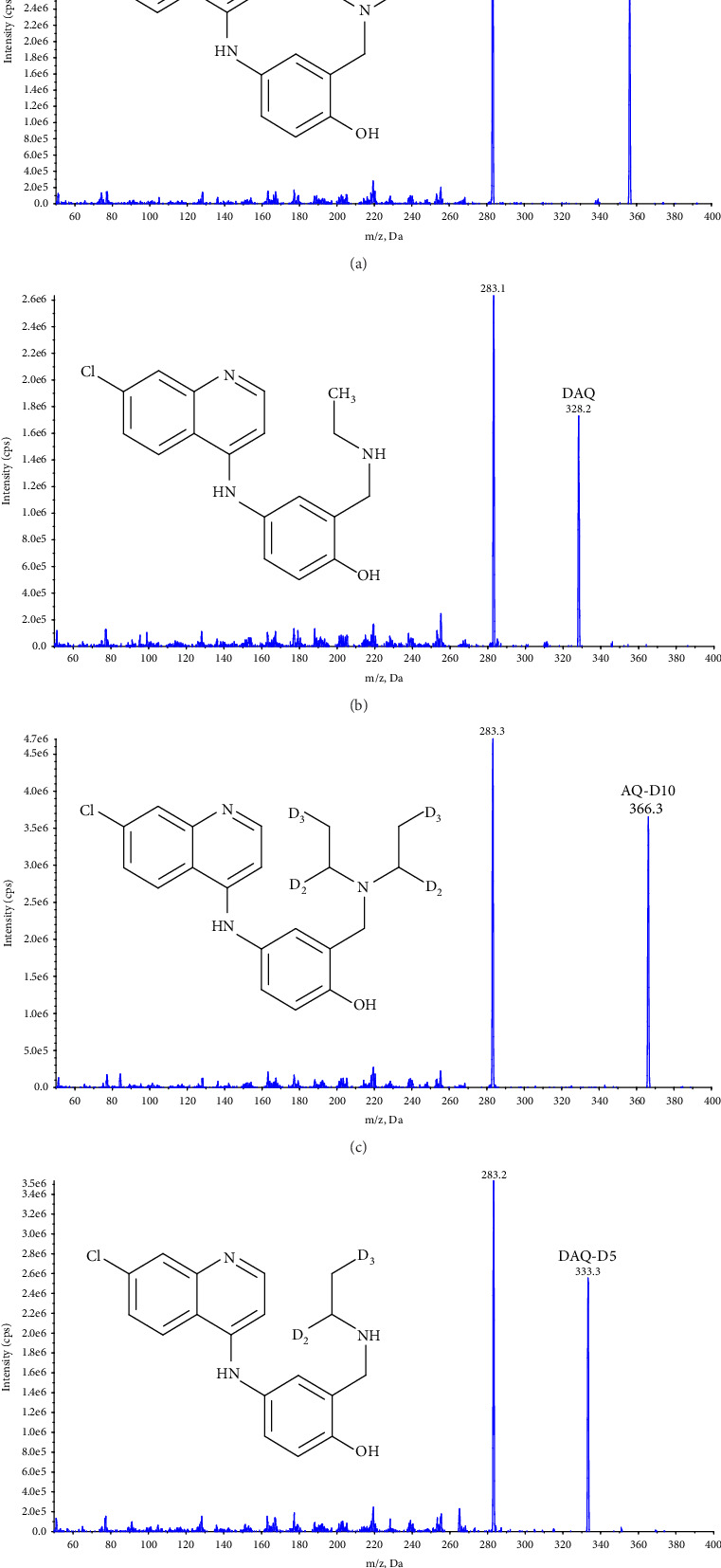
Cumulative collision energy scan and the product ion formed of (a) AQ (m/z 356.4), (b) DAQ (m/z 328.2), (c) AQ-D10 (m/z 366.3), and (d) DAQ-D5 (m/z 333.3) [25].

Quantification was performed using selected reaction monitoring (SRM) for the transitions m/z 356.4 ⟶ 283.2 and 366.3 ⟶ 283.3 for AQ, and AQ-D10, and 328.2 ⟶ 283.1 and 333.3 ⟶ 283.2 for DAQ and DAQ-D5, respectively (Figure 1). All transitions used collision energy of 29 V.

### 2.6. Method Validation

Method validation was performed according to the US Food and Drug Administration (FDA), 2018 [26] and International Council for Harmonization (ICH), 2022 guidelines [27].

Weighted (1/*x* and 1/*x*^2^) and nonweighted linear and quadratic regression models were evaluated for the calibration curve. The best performing model was chosen based on the lowest total sum of relative residuals of back-calculated concentrations compared with the nominal concentrations of the calibration curves and QC samples (equation (1)), calculated and summarized from four individual runs [28]. The best performing model was also evaluated based on accuracy and precision of back-calculated concentrations of the calibration curves and QC samples to verify that the performance met regulatory guidelines.

(1) $$\begin{array}{c} \text{Relative residual}=\frac{\left|\text{Predicted conc}.-\text{Nominal conc}.\right|}{\left(\text{Nomical conc}.\right)}. \end{array}$$

Accuracy and precision were calculated as the mean relative error (%) and coefficient of variation (%CV), respectively. A single factor ANOVA was used for precision calculations (intrabatch, interbatch, and total-assay variability). Both were determined by analyzing five replicate samples over four independent runs at LLOQ, ULOQ, as well as QC samples at three concentrations.

The overcurve samples were used to assess the ability to dilute samples (1:5 and 2:5 dilutions) above the ULOQ. Dilution integrity was investigated by analyzing five replicates at 2-3 × ULOQ for AQ and DAQ. Selectivity was assessed using blank healthy human EDTA DBS from six donors. Potential interference from endogenous compounds and their separation from AQ and DAQ were evaluated.

Sensitivity was evaluated by comparing the signal intensity of AQ and DAQ at LLOQ in extracted DBS samples compared with extracted blank DBS sample.

Specificity was evaluated by assessing carryover, interference from coadministered drugs during postcolumn infusion, and interference from internal standards and analytes. Carryover effects were investigated by injecting five ULOQ samples followed by three blank samples. A signal higher than 20% of LLOQ in the injected blank samples would indicate carryover. For postcolumn infusion experiments, a mix of AQ, DAQ, and their SIL solutions at 20 ng/mL were used. Five possible coadministered drugs were injected as neat solutions at or above their therapeutic range while performing postcolumn infusion; ARS (546 ng/mL), artemether (373 ng/mL), lumefantrine (4346 ng/mL), SUD (32,700 ng/mL), and PYM (32,700 ng/mL). Potential interference between analytes and internal standards was evaluated by assessing the impact of internal standards (AQ-D10, 0.86 ng/mL and DAQ-D5, 0.86 ng/mL) on the analytes at LLOQ. In addition, potential interference from analytes on the internal standards was assessed at the highest analyte concentration at ULOQ (without their respective SIL), focusing on their impact on the internal standards with respect to retention times and signal intensity. Specificity was further evaluated using blank DBS samples, both with and without internal standards, to confirm the absence of interference.

Quantitative matrix effects were evaluated using DBS from six different EDTA donors. The extracted blank DBS from each donor were spiked with analyte and SIL (i.e., postspiked blank extracted samples) and compared with AQ, DAQ, and their SIL solutions to determine matrix factors and normalized matrix factors, respectively, to investigated potential ion suppression or enhancement caused by the matrix.

Process efficiency and absolute recovery were determined by five replicates of extracted QC samples compared with neat solution and postspiked blank DBS extracted samples.

Stability of AQ and DAQ in DBS was investigated by exposing the samples to five freeze and thaw cycles. The samples were frozen at −80°C for 24 h for the first freeze cycle and 12–24 h for the following freezing cycles with in-between thawing at RT for 2-3 h. Short-term stability at RT (22°C) and at refrigerator temperature (4°C) was investigated at 4, 24, and 48 h. Long-term stability of spiked samples in storage condition was evaluated for up to 10 years (−80°C) and for 15 months when stored at RT. The stability of the analytes during the extraction process was evaluated for the following parameters: stability of extracted samples in extraction solution at 4°C for 24 h and the stability in injection-ready samples in the LC autosampler at 4°C for up to 72 h.

Stability tests of AQ and DAQ in the presence of coadministered drugs were conducted using blood spiked with separate spiking for each coadministered drug (ARS and artemether at 10 μg/mL and lumefantrine at 2.5 μg/mL), except for SUD and PYM, which were spiked together at 10 μg/mL before spotting on filter paper. The tests included five freeze-thaw cycles, short-term stability at RT 22°C and 4°C for up to 24 h, and long-term stability at both RT 22°C and 4°C for up to 34 days, with spiked samples stored at −80°C serving as the reference for comparison.

### 2.7. DBS-Specific Tests

Spiked blood applied on Whatman 31 ET Chr paper was used to test different drying conditions of DBS in very high humidity conditions 27°C–32°C and 88%–92% relative humidity (RH) and at dry conditions in a dry cabinet 20°C, 20% RH. After spotting (50 μL), the blood spots were left to dry for 2 h at RT and then transferred to different storage conditions e.g. slow/fast drying for 1–6 h in low/high humidity conditions between 40% and 80% RH. In wet tropical conditions, for example, rainy season, drying in high humid conditions is not possible. To simulate these conditions, including an alternative drying approach, after blood spotting, the wet spot was directly transferred to a plastic bag and a desiccant was added to aid the drying process and the bag was then stored at room temperature for the spot to dry in the plastic bag with the assistance of a desiccant. The DBS samples were left for 2 weeks in different environments e.g. high humid (> 80% RH, 30°C), at room temperature (about 50% RH, 22°C) and store at −20°C. These dried stability samples were then analyzed against frozen (−80°C) reference samples.

The impact of using alternative filter papers with similar properties as Whatman 31 ET Chr was evaluated. Two QC levels of AQ and DAQ spotted on Whatman (DMPK-C, 903 Protein saver and 3 MM Chr) were comparing against Whatman 31 ET Chr as reference [24].

The impact of hematocrit in DBS sample was evaluated using the Whatman 31 ET Chr paper. After EDTA blood centrifugation (2000 ×g, 10 min) plasma was added or removed to achieve an erythrocyte volume fraction of 20% and 60%. Blood was then spiked and spotted (100 μL) and allowed to dry completely before storage at −80°C with a desiccant bag. Subsequent analysis used five punches from the same spot at the center and five punches close to the edge of the DBS for each hematocrit and QC level combination [29, 30]. The average concentration of each hematocrit level was compared to the reference obtained from healthy individuals (∼35–50% hematocrit). This comparison was calculated as (sample-reference) divided by reference, aiming to assess any impact of hematocrit and punch position. The deviation should be within ±15% of the nominal concentration.

### 2.8. Clinical Applicability

The validated DBS method was implemented for analysis of DBS patient samples in a study to describe the population pharmacokinetics of AQ and DAQ in the setting of seasonal malaria chemoprevention (SMC) [31].

## 3. Result and Discussion

### 3.1. Method Validation

**Figure 2:**
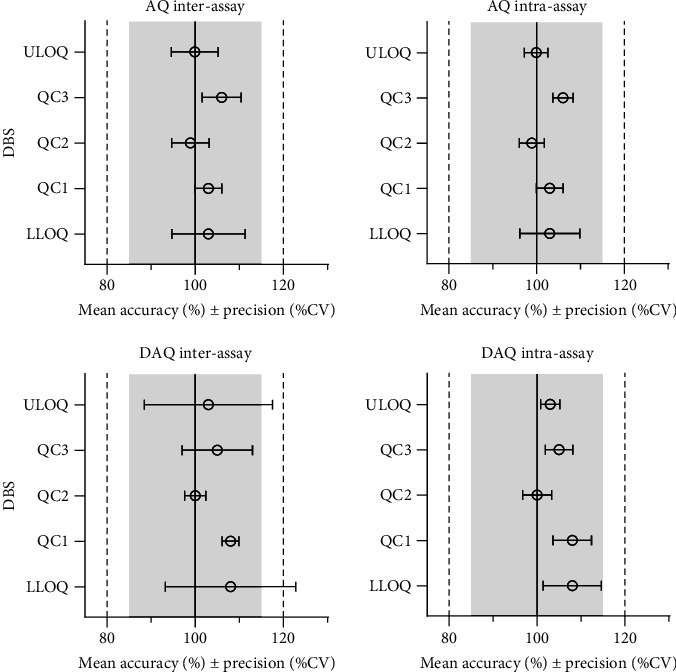
Accuracy and precision of QCs, LLOQ, and ULOQ in EDTA DBS samples. The circle (○) is mean accuracy (%) and the error bars is inter-, intra-assay precision (ANOVA).

The most suitable regression model for AQ and DAQ in DBS samples was a quadratic regression with 1/*x*^2^ weighting, resulting in the best prediction of back calculated values for both the standard curve and QC samples [28, 32]. Quadratic regression with 1/*x*^2^ weighting, resulted in the lowest total sum of relative residuals for both AQ (5.7) and DAQ (7.6), and linear regression with 1/*x*^2^ weighting resulted in a larger sum of relative residuals for AQ (6.5) and DAQ (9.2). Both linear and quadratic regression with 1/*x* weighting resulted in worse predictive performance, demonstrated by larger sums of relative residuals. Non-weighted regression showed a very poor performance at low concentrations and an overall predictive performance that did not confirm to validation guideline criteria for accuracy and prediction (> 15%). The final, best performing, quadratic regression model with 1/*x*^2^ weighting showed acceptable accuracy and precision and each calibration curve showed a high correlation coefficient (*r* > 0.997). This calibration curve range of AQ (2.03–459 ng/mL) and DAQ (3.13–1570 ng/mL) covers the therapeutic range that is commonly found in blood [8] and accommodates the slow elimination of DAQ [8, 11, 33–37]. The accuracy of AQ and DAQ ranged from 99% to 108%, while inter-day and intra-day precision (% CV) varied from 2% to 14% across the LLOQ, ULOQ, and QC levels (Figure 2 and Supporting Information Table S3).

Dilution integrity was assessed using over-curve samples (AQ 1837 ng/mL and DAQ 4901 ng/mL) with five replicates at 1:5 and 2:5 dilutions, resulting in AQ accuracy of 94% and 96%, and DAQ accuracy of 99% and 104% respectively. For AQ and DAQ, precision was within 5%.

All blank sources (six different donors) was free from interfering signals, demonstrated by a total contribution of < 20% of LLOQ concentrations and ≤ 5% of their SIL response. This result showed that the validated DBS method had high selectivity without interference from the different individual donor matrices (Supporting Information Figure S1).

**Figure 3:**
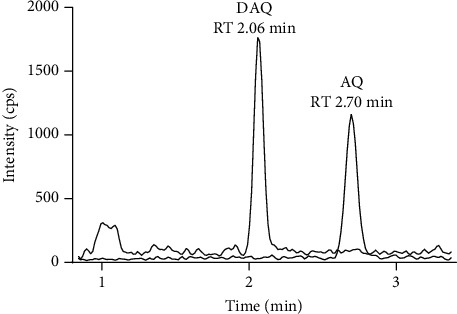
Extracted ion chromatogram of a DBS sample at LLOQ, containing 2.03 ng/mL AQ and 3.13 ng/mL DAQ. The LC-pump is switching to a washout with mobile phase B at 2.2 min, however the washout solvent does not reach the column until about 3.3 min, due to the void volume in the LC-system that includes a LC-pump pulse dampener.

Sensitivity of the method was evaluated as signal intensity of extracted DBS samples at LLOQ compared with a blank DBS sample. AQ/DAQ was clearly distinguishable from the blank sample with an average signal-to-noise ratio > 10 (Figure 3). These results indicated that even a small blood volume from a DBS (equivalent to approximately 15 μL blood in the punched-out discs) and a small LC injection volume could achieve adequate sensitivity.

Carryover specificity was assessed by injecting three extracted blank filter papers and a blank mobile phase after five ULOQ replicates. No detectable carryover of AQ/DAQ or their SIL (AQ-D10/DAQ-D5) was observed.

AQ, DAQ and their SIL were well separated within the 2.2 min isocratic portion of the assay, followed by a washout period with mobile phase B. The washout period was used to flush out any strongly retained compounds that might otherwise accumulate on the column, reducing the column performance over time or potentially co-eluting with the analytes in subsequent injections, causing matrix effects [38, 39]. Therefore, no carryover was detected under these conditions.

Interference from co-administered drugs was assessed by post-column infusion and injection of extracted blank DBS and five possible co-administered drug solutions at or above the therapeutic range in the treatment or prevention of malaria [2, 3]; ARS (546 ng/mL), artemether (373 ng/mL), lumefantrine (4,346 ng/mL), SUD (32,700 ng/mL), and PYM (32,700 ng/mL). These potentially coadministered drugs did not produce any interfering peaks or suppression or enhancement regions around the retention time of AQ or DAQ (Supporting Information Figure S2). Internal standard interference testing (AQ-D10 0.86 ng/mL, DAQ-D5 0.86 ng/mL) showed no interference at AQ and DAQ retention times (Supporting Information Figure S3). Analytes at ULOQ, showed no interference to their SIL, with all acceptance criteria met (interference peaks ≤ 20% of AQ/DAQ at LLOQ and ≤ 5% of SIL response) (Supporting Information Figure S4). In addition, blank DBS samples with and without internal standards confirmed the absence of interference (Supporting Information Figure S5).

Quantitative matrix effects were evaluated using blank EDTA DBS samples from six donors, post-spiked and compared with neat reference solutions (Table 1). All matrix factors were close to 1 with low variation (1.03–1.11), indicating slight signal enhancement of AQ, DAQ, and their SIL in DBS samples compared to solution. The normalized matrix effects, expressed as AQ/AQ-D10 and DAQ/DAQ-D5 ratios, were close to 1, indicating no significant ion suppression or enhancement at AQ, DAQ, and SIL retention times.

The process efficiency (extracted DBS/neat solution) and absolute recovery (extracted DBS/blank DBS postspiked) at low and high QC levels yielded similar results, ranging from 54% to 68% for both AQ and DAQ. Both of their SIL demonstrated high recoveries, ranging from 78% to 90%.

The lower absolute recovery of AQ and DAQ in the DBS sample compared to their SIL might be due to AQ and DAQ being harder to extract from a dried spot on chromatography paper, thereby affecting extraction efficiency. The cellulose fibers (β-anhydroglucose units with dominant hydroxyl groups) in Whatman 31 ET Chr chromatography paper could possibly be interacting with AQ and DAQ structures during the application and drying process [40]. However, their SIL, AQ-D10 and DAQ-D5, were added in extraction solution and would not bind to the paper to the same extent. Therefore, AQ-D10 and DAQ-D5 recoveries were not dependent on chromatography paper but rather the extraction technique utilized (Phree phospholipid removal technique), and therefore providing better absolute recovery than AQ and DAQ.

The five freeze/thaw cycle stability, short term stability at 22°C, 4°C and −20°C (112, 146 and 184 h), extracted sample stability (stored extracted sample at 4°C for 24 h) and heat stability stress test (60°C, ∼1.5 h) were all stable. AQ and DAQ were stable for at least 48 h in the LC autosampler at 4°C. Accuracy and precision (CV%) for stability samples were all within the range of the acceptance criteria (±15%) (Supporting Information Table S1).

The evaluation of long-term storage stability was dependent on actual storage condition of spiked samples. AQ and DAQ in DBS were stable for at least 10 years when stored at −80°C. However, at room temperature (and below 20% RH when stored with a desiccant) both AQ and DAQ exhibited instability, showing about 15% degradation after 20 months and 40%–50% degradation after 7 years (Supporting Information Table S2). It has been difficult to determine whether the observed instability of AQ and DAQ is due to factors beyond moisture and common degradation pathways or an increased difficulty in extraction of older DBS. However, during shorter storage times, both AQ and DAQ were found to be stable in DBS at RT for at least 15 months (∼20% RH, 22°C). These results indicate analytes can be considered sufficiently stable for short-term storage under all tested conditions, but for long-term stability, DBS should be stored at −80°C to prevent analyte degradation.

Furthermore, in the presence of coadministered drugs (ARS, artemether, lumefantrine, SUD, and PYM), stability tests showed that AQ and DAQ remained stable through five freeze/thaw cycles at −80°C and maintained stability for at least 24 h at both 22°C and 4°C. In addition, AQ and DAQ exhibited stability for at least 34 days at 22°C and 4°C, even with other drugs present. All accuracy and precision values remained within ±15% (Table 2).

### 3.2. DBS-Specific Tests

DBS specific short-term stability was evaluated by simulating initial drying and storage condition for DBS samples that might be encountered in real field conditions, e.g., dry/wet season. The test conditions involved slow/fast drying in low/high humidity conditions (40%–80% RH) over a period of up to 2 weeks, after which the drug measurements were conducted. Results indicated that drying at RT for 2 h and subsequent storage at approximately 50% RH and 22°C, or in a freezer at −20°C, within a plastic bag containing desiccant, did not result in the loss of AQ and DAQ (Table 3). However, loss was observed under high humidity drying and storage conditions (> 80% RH, 30°C). In these conditions, the measurement of AQ and DAQ in the DBS sample was impacted, leading to decreased drug recovery and accuracy values ranging from 58% to 69%. Furthermore, if a freshly spotted sample was immediately transferred to a plastic bag with a desiccant for drying under typical room humidity conditions (∼50% RH, 22°C), this resulted in an accuracy range of 71%–85%. After the test, it was found that the desiccant had become saturated and may not have fully dried the sample, potentially impacting the results. This indicated that if the blood spot does not achieve complete dryness at very high humidity or inside plastic bag conditions, and this can affect its stability. In order to avoid stability issues and to prevent mold/fungal growth, DBS samples require low humidity (< 40% RH) of the surrounding environment for rapid drying and long-term storage, and the samples should dry completely before long-term storage in plastic bags in the presence of a desiccant. A way to limit the degradation of samples taken during humid conditions in the field is recommended to place the blood spots in a plastic bag with enough desiccant to dry the DBS spot and for it to stay dry or to use a dry cabinet to assist the drying process. Ordinary plastic bags are not moisture proof and some moisture can permeate through them over time. For optimal protection, it is best to use moisture barrier bags. Thus, storage of AQ and DAQ as DBS samples does not require a refrigerator or freezer, which makes the use of this type of blood sample convenient and can reduce the cost and increase the flexibility of the study site, particularly in low resource settings.

Other chromatography papers with similar properties to the Whatman 31 ET Chr, e.g., Whatman DMPK-C and 903 Protein saver gave comparable accuracy and precision and may be used as alternatives for DBS sampling and quantification [24]. However, a thinner paper like the Whatman 3 MM Chr chromatography paper is not recommended due to differences in blood absorption properties. This thin paper provided a noncircular and a wider blood spot, resulting in a bias of blood volume when compared with the thicker papers after punched out from the blood spot, and this affected the AQ and DAQ measurement (Table 4).

Normal hematocrit levels in healthy individuals range between 35% and 50% and differs from clinical malaria patients who often show low levels due to hemolysis of malaria-infected red blood cells. Patients with severe malaria can display very low levels (< 20%) of hematocrit [41]. The blood spot size will differ when comparing low and high hematocrit levels, where smaller blood spots are produced in blood with high hematocrit resulting in a higher volume of blood obtained per punched disc. Conversely, at lower hematocrit levels (e.g., 20%), blood spots tend to be larger because there are fewer red blood cells relative to plasma. The main purpose of this experiment was to investigate if differences in patient sample hematocrit affected the accuracy of drug quantification. Measurements of AQ and DAQ from 100 μL spot sizes indicate that low (20%) and high (60%) hematocrit levels influence the quantification of AQ and DAQ. Low hematocrit underestimate the quantification results (accuracy of 84%–93%), while high hematocrit leads to a slight overestimation of results (accuracy of 100%–107%) across the two QC levels when compared with blood with a normal hematocrit (Table 5). Therefore, sample hematocrit should be considered when interpreting the results from patient samples. However, the location of punches (center or edge) in DBS samples with varying hematocrit levels did not significantly impact AQ and DAQ quantification. The differences between center and edge punches were within ±9%, which falls within the method's normal variation (Table 6). This indicates that punch location does not significantly affect the accuracy of AQ and DAQ measurements.

### 3.3. The Validated DBS Method Applicability to Clinical Routine Analysis

**Figure 4:**
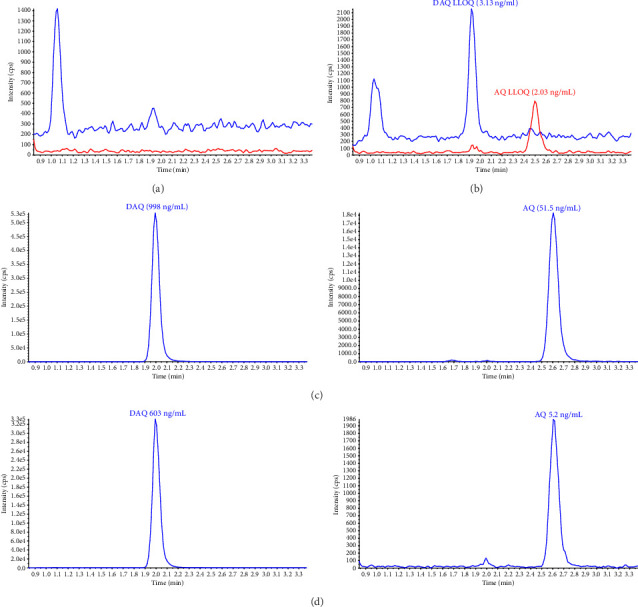
Extracted ion chromatograms of AQ and DAQ in blank and LLOQ DBS extractions, along with routine sample analyses from a seasonal malaria chemoprevention study [30]. The upper section: (a) extracted blank DBS and (b) extracted LLOQ (AQ 2.03 ng/mL and DAQ 3.13 ng/mL). The middle section: (c) patient chromatogram at 4 h after first dose (AQ: 51.5 ng/mL and DAQ: 998 ng/mL). The lower section: (d) patient chromatogram at 7 days after first dose (AQ: 5.2 ng/mL and DAQ: 603 ng/mL). The patient sample represent a child in the age group 12–59 months, receiving three standard doses of AQ (150 mg) over three days in a malaria-endemic setting.

The implementation of the validated method for high-throughput routine sample analysis proved effective in assessing the protective effectiveness of seasonal malaria chemoprevention (SMC) in children aged 3–59 months in a malaria-endemic setting [32]. A total of 1600 DBS samples were analyzed, with 37.3% falling within the validated range for AQ and 67.6% for DAQ. The percentage of DBS samples above ULOQ and below LLOQ for AQ were 1.4% and 61.3%, respectively. Similarly, for DAQ, these percentages were 2.1% and 30.3%, respectively. In this study, samples were collected for up to 28 days after drug administration, which explains the relatively high proportion of samples that were below the LLOQ. Chromatograms of blank and LLOQ extractions as well as real study samples are presented in Figure 4. The method demonstrated good precision and accuracy, with all 204 QC samples at each concentration level meeting the acceptance criteria of ±15% deviation. Accuracy for the low, medium, and high QC samples was 90%, 92%, and 94% for AQ and 90%, 92%, and 91% for DAQ. Relative standard deviations (%CV) were below 6%, confirming the method's reliability and suitability for assessing these drugs in patient samples to evaluate the efficacy of malaria chemoprevention in a pediatric population.

## 4. Conclusion

The sample extraction technique presented here used the phospholipid removal technology to remove residual phospholipids that could, otherwise, contribute to matrix effects. This extraction technique is a fast and simple approach to quickly remove proteins and phospholipids from DBS samples while still maintaining precise and accurate data. The clean sample extraction technique combined with automated sample preparation makes this method high-throughput and suitable for large batch analysis. This assay passed all validation criteria and there was no detectable carryover. The developed DBS method is suitable for pharmacokinetic studies where capillary finger/heel prick sampling, with a small capillary blood volume of approximately 50 μL, is carried out. This field-adapted sampling technique and the small sample volume enables pharmacokinetic evaluations in underserved populations, such as small children, and malaria field studies in resource-limited settings.

## Figures and Tables

**Table 1:** Quantitative matrix effects (matrix factor) of AQ and DAQ in DBS samples.

Donor	AQ				DAQ			
	QC1	SIL (QC1)	QC3	SIL (QC3)	QC1	SIL (QC1)	QC3	SIL (QC3)
1	1.04	1.03	1.05	1.04	1.07	1.06	1.06	1.05
2	1.06	1.04	1.03	1.03	1.09	1.06	1.07	1.05
3	1.06	1.05	1.04	1.04	1.09	1.07	1.08	1.05
4	1.08	1.08	1.05	1.04	1.11	1.10	1.10	1.05
5	1.05	1.06	1.06	1.06	1.09	1.09	1.09	1.05
6	1.06	1.07	1.04	1.04	1.08	1.06	1.07	1.06

Average	1.06	1.06	1.04	1.04	1.09	1.07	1.08	1.05
SD	0.0139	0.0190	0.0101	0.0090	0.0120	0.0170	0.0132	0.0053
CV (%)	1.3	1.8	1.0	0.9	1.1	1.6	1.2	0.5

*Note:* AQ = amodiaquine, DAQ = desethylamodiaquine, SIL = stable isotope-labeled internal standard. All incubations were conducted in triplicates using 50 μL EDTA DBS.

Abbreviations: %CV = Percent coefficient of variation, QC = quality control, SD = Standard deviation.

**Table 2:** Stability of AQ and DAQ in DBS samples with coadministered antimalarial drugs.

Stability	AQ				DAQ			
	QC1		QC3		QC1		QC3	
	Accuracy (%)	Precision (%CV)	Accuracy (%)	Precision (%CV)	Accuracy (%)	Precision (%CV)	Accuracy (%)	Precision (%CV)
RT (22°C), 24 h								
+ARS	93.4	1.7	91.2	2.2	104	3.8	88.7	2.8
+ARM	90.5	2.7	90.8	2.8	100	2.9	86.9	2.3
+LF	90.0	4.1	87.3	6.6	103	1.8	86.5	3.1
+SUD–PYM	92.8	2.4	90.4	1.2	102	0.5	87.9	0.5
Refrigerator (4°C), 24 h								
+ARS	92.0	5.0	87.0	2.9	103	1.7	87.9	2.7
+ARM	88.8	1.6	89.7	2.8	96.2	3.0	88.4	1.5
+LF	93.2	1.8	92.1	1.2	99.0	1.3	88.7	1.3
+SUD–PYM	90.1	5.6	90.8	3.2	99.4	2.8	87.9	3.3
Freeze-thaw (−80°C), 5 cycles								
+ARS	93.7	1.0	96.1	3.0	98.4	5.0	95.3	2.3
+ARM	93.0	4.4	93.9	2.0	100	3.9	92.2	2.8
+LF	96.5	2.4	98.5	1.2	97.2	4.2	95.7	2.9
+SUD-PYM	97.9	3.0	96.6	0.9	101	2.7	96.2	2.7
Long-term stability: RT^∗^ (∼20% RH, 22°C), 34 days								
+ARS	98.7	6.0	90.4	4.1	106	3.5	93.5	3.29
+ARM	90.4	1.2	88.9	1.5	102	3.2	96.5	1.99
+LF	91.2	2.5	88.2	3.3	104	5.0	89.7	1.77
+SUD–PYM	90.8	3.1	89.1	2.1	105	3.8	99.1	0.769
Long-term stability: refrigerator (4°C), 34 days								
+ARS	93.7	2.7	96.1	2.88	109	2.9	108	2.7
+ARM	91.6	3.6	92.1	2.63	107	2.0	105	1.5
+LF	99.8	1.2	95.9	1.77	115	5.4	108	6.9
+SUD–PYM	97.9	1.8	92.8	1.27	115	2.9	104	1.9

*Note:* AQ = amodiaquine, ARS = artesunate, ARM = artemether, DAQ = desethylamodiaquine, LF = lumefantrine, SUD–PYM = sulfadoxine–pyrimethamine. All incubations were conducted in triplicates, using 50 μL EDTA DBS.

Abbreviations: °C = degrees celsius, %CV = percent coefficient of variation, QC = quality control, RH = relative humidity, RT = room temperature.

^∗^Stored at room temperature in a dry cabinet (∼20% RH, 22°C).

**Table 3:** Stability of AQ and DAQ at different drying conditions over a period of up to 2 weeks.

Drying condition	Storage condition	AQ		DAQ	
		Accuracy ± precision (%)		Accuracy ± precision (%)	
		QC1	QC3	QC1	QC3
RT	∼50% RH, 22°C	102 ± 1.3	96.3 ± 1.0	111 ± 10.6	100 ± 4.2
RT	−20°C	96.1 ± 4.9	92.3 ± 3.1	102 ± 4.3	97.7 ± 3.5
High humid (> 80% RH)	> 80% RH, 30°C	58.0 ± 3.4	60.1 ± 2.2	61.2 ± 6.8	68.8 ± 1.4
Plastic bag with desiccant	∼50% RH, 22°C	80.1 ± 6.7	71.2 ± 8.2	85.3 ± 5.6	76.3 ± 5.5

*Note:* AQ = amodiaquine, DAQ = desethylamodiaquine. All incubations were conducted in triplicates, using 50 μL EDTA DBS.

Abbreviations: RT = room temperature, RH = relative humidity, QC = quality control.

**Table 4:** Type of filter papers for AQ and DAQ.

Type of filter paper	AQ		DAQ	
	Accuracy ± precision (%)		Accuracy ± precision (%)	
	QC1	QC3	QC1	QC3
31ET Chr (reference)	101 ± 4.5	96.2 ± 4.9	95.8 ± 7.2	94.9 ± 2.5
DMPC-K	113 ± 6.6	104 ± 1.9	105 ± 2.6	105 ± 4.1
903 protein	115 ± 2.5	106 ± 4.5	103 ± 2.5	105 ± 5.2
3 MM Chr	75.3 ± 5.3	68.2 ± 4.4	74.7 ± 7.7	71.0 ± 7.0

*Note:* AQ = amodiaquine, DAQ = desethylamodiaquine. All incubations were conducted in triplicates, using 50 μL EDTA DBS.

Abbreviation: QC = quality control.

**Table 5:** Impact of low and high hematocrit level, compared with the normal value (42%), on AQ and DAQ measurements at center punch.

Drug	Sample	Differences	
		20% HCT (%)	60% HCT (%)
AQ	QC1	−15.6	0.3
	QC3	−10.2	5.3

DAQ	QC1	−13.5	2.0
	QC3	−6.6	6.9

*Note:* AQ = amodiaquine, DAQ = desethylamodiaquine, HCT = hematocrit. All incubations were conducted in five replicates (*n* = 5) using 100 μL EDTA DBS.

Abbreviations: DBS = dried blood spot, QC = quality control.

**Table 6:** Difference in AQ and DAQ measurements between center and edge punch locations at different hematocrit levels.

Hematocrit level (%)	Difference: center vs. edge			
	AQ		DAQ	
	QC1 (%)	QC3 (%)	QC1 (%)	QC3 (%)
20	−4.2	−2.6	−6.8	−8.6
42 (normal value)	−3.5	−3.9	−3.9	−4.7
60	1.6	0.6	−2.1	−0.6

*Note:* AQ = amodiaquine, DAQ = desethylamodiaquine. All incubations were conducted in five replicates (*n* = 5) using 100 μL EDTA DBS.

Abbreviations: DBS = dried blood spot, QC = quality control.

## Data Availability

The data used to support the findings of this study are included within the article.

## Supporting Information

The data provided in the Supporting Information, which also support this study, include Table S1: Short-term stability of AQ and DAQ in DBS samples, Table S2: Long-term stability of AQ and DAQ in DBS samples, Table S3: Accuracy and precision of AQ and DAQ extracted from DBS samples, Figure S1: Method selectivity, confirming the absence of interference from different individual donor matrices, Figure S2: Postcolumn infusion data for matrix effect evaluation, Figure S3: Potential interference from internal standard to analyte, Figure S4: Potential interference from analyte to internal standard, and Figure S5: Method specificity by confirming the absence of endogenous interference from blank DBS extraction with and without internal standards.
