# Supplementary material for: A LC-MS/MS Assay for Quantification of Amodiaquine and Desethylamodiaquine in Dried Blood Spots on Filter Paper
Source: Int J Anal Chem. 2025 Jun 5;2025:5130424. doi: 10.1155/ianc/5130424 (PMC12163217; doi:10.1155/ianc/5130424)
Supplement: Supporting Information — Additional supporting information can be found online in the Supporting Information section. [file 5130424.f1.pdf]

Supplementary material

A LC-MS/MS assay for quantification of amodiaquine and desethylamodiaquine in dried blood spots on filter paper

Natpapat Kaewkhao[1], Joel Tarning[1,2], Daniel Blessborn[1,2]*

[1]Mahidol Oxford Tropical Medicine Research Unit, Faculty of Tropical Medicine, Mahidol University, Bangkok, Thailand.

[2]Centre for Tropical Medicine & Global Health, Nuffield Department of Clinical Medicine, University of Oxford, Oxford, UK

*Author for correspondence: natpapat@tropmedres.ac

The Supplementary Materials for this study include:

- Table S1. Short-term stability data of amodiaquine (AQ) and desethylamodiaquine (DAQ) in DBS samples.
- Table S2. Long-term stability data for amodiaquine (AQ) and desethylamodiaquine (DAQ) in DBS samples.
- Table S3. Accuracy of amodiaquine (AQ) and desethylamodiaquine (DAQ) in DBS samples across four independent runs at LLOQ, ULOQ, and three QC concentrations.
- Figure S1. Extracted ion chromatograms of amodiaquine (AQ) and desethylamodiaquine (DAQ) at LLOQ overlaid with six different donors (A-F) in DBS samples.
- Figure S2. Extracted ion chromatograms of amodiaquine (AQ), desethylamodiaquine (DAQ), and their stable isotope-labelled internal standards (AQ-D10 and DAQ-D5) during post-column infusion, illustrating matrix effects with five co-administered drugs.

- Figure S3. Potential interference, evaluated by assessing the impact of internal standards (AQ-D10 0.86 ng/ml, DAQ-D5 0.86 ng/ml) on the analytes.
- Figure S4. Potential interference, evaluated by assessing the impact of the highest analyte concentration at ULOQ (without their respective SIL) on the internal standards at their retention times.
- Figure S5. Chromatography of blank DBS extraction with and without internal standards, demonstrating specificity by confirming the absence of endogenous interference.

TABLE S1: Short term stability of amodiaquine (AQ) and desethylamodiaquine (DAQ) in DBS sample.

Stability	AQ				DAQ			
	QC1		QC3		QC1		QC3	
	Accuracy (%)	Precision (%CV)	Accuracy (%)	Precision (%CV)	Accuracy (%)	Precision (%CV)	Accuracy (%)	Precision (%CV)
Room temperature in dry cabinet (~50%RH, 22°C)								
112 hr	99.0	3.8	97.4	3.2	103	5.4	95.0	5.3
146 hr	95.3	12.5	96.4	3.9	96.5	4.0	94.7	5.1
184 hr	108	9.7	96.7	5.9	101	10.3	100	4.4
Refrigerator (4°C)								
112 hr	88.5	6.7	91.6	2.2	98.0	5.1	92.5	3.3
146 hr	92.3	2.3	90.4	0.9	92.8	6.8	89.7	0.5
184 hr	102	3.8	94.5	2.9	92.8	5.7	94.2	6.0
Freezer (-20°C)								
112 hr	103	3.4	94.1	3.6	103	4.6	94.7	3.9
146 hr	93.7	6.4	95.2	1.2	96.7	6.1	99.2	3.1
184 hr	95.4	5.9	97.4	2.7	94.5	5.0	93.7	4.8
Freeze-Thaw (-80°C)								
Cycle 1	105	5.2	94.6	3.8	95.1	4.8	97.7	2.2
Cycle 5	106	8.6	94.9	2.1	93.0	8.3	94.7	4.4
Autosampler (4°C, 48 hr)	99.0	5.4	93.5	3.5	91.7	2.2	100	3.0
Re-extracted (4°C, 24 hr)	97.0	6.5	94.6	1.0	101	7.0	97.0	2.5
Heat stability (60°C, 1.5 hr)	91.4	3.1	94.2	2.8	94.6	5.8	94.7	2.0

%CV: Percent coefficient of variation; QC: Quality control; RH: Relative humidity.
All incubations were conducted in triplicates, using 50 µl EDTA DBS.

TABLE S2: Long term stability of amodiaquine (AQ) and desethylamodiaquine (DAQ) in DBS sample.

Stability	AQ				DAQ			
	QC1		QC3		QC1		QC3	
	Accuracy (%)	Precision (%CV)	Accuracy (%)	Precision (%CV)	Accuracy (%)	Precision (%CV)	Accuracy (%)	Precision (%CV)
Room temperature in dry cabinet (~20%RH, 22°C)								
5 months	102	8.1	93.5	3.1	101	3.1	85.2	4.1
15 months	85.1	4.2	86.8	3.0	88.3	2.3	87.0	2.5
20 months	84.3	1.7	85.9	0.7	86.0	3.2	84.6	3.6
7 years	54.7	8.4	48.1	3.9	59.3	7.2	47.4	3.2
8 years	49.9	5.6	44.9	2.8	58.0	3.6	49.9	2.8
10 years	61.3	3.2	51.7	3.2	67.0	3.9	58.8	3.0
Freezer (-80°C)								
5 months	105	4.3	105	5.8	106	4.8	96.6	3.9
10 years	98.2	4.7	97.0	2.8	89.6	2.3	88.5	3.1

%CV: Percent coefficient of variation; QC: Quality control; RH: Relative humidity.
All incubations were conducted in five replicates (n=5), using 100 µl EDTA DBS.

TABLE S3: Accuracy and precision of amodiaquine (AQ) and desethylamodiaquine (DAQ) extracted from DBS samples.

AQ					DAQ				
Sample (ng/mL)	Measurement (ng/mL)	Accuracy (%)	Precision (%CV) inter-assay	intra-assay	Sample (ng/mL)	Measurement (ng/mL)	Accuracy (%)	Precision (%CV) inter-assay	intra-assay
LLOQ (2.03)	2.10	103	8.1	6.7	LLOQ (3.13)	3.37	108	13.7	6.1
ULOQ (459)	458	99.9	5.3	2.7	ULOQ (1570)	1617	103	14.1	2.1
QC1 (6.12)	6.3	103	2.9	3.0	QC1 (9.75)	10.5	108	1.7	4.1
QC2 (184)	182	98.9	4.3	2.9	QC2 (603)	604	100	2.4	3.3
QC3 (383)	405	106	4.2	2.2	QC3 (1312)	1380	105	7.6	3.0

%CV: Percent coefficient of variation; QC: Quality control; LLOQ: Lower limit of quantification; RH: Relative humidity; SD: Standard deviation; ULOQ: Upper limit of quantification. All incubations were conducted in five replicates (n=5), using 50 µl EDTA DBS.

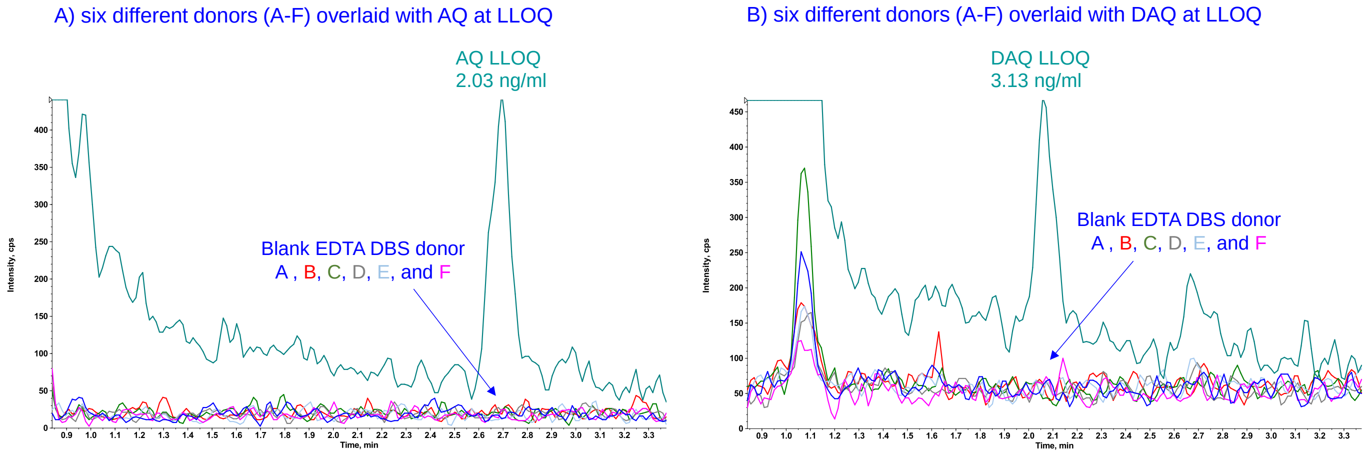

FIGURE S1: Overlay of the extracted ion chromatogram for A) amodiaquine (AQ) at LLOQ of 2.03 ng/mL and B) desethylamodiaquine (DAQ) at 3.13 ng/mL, showing data from six different donors: A (blue), B (red), C (green), D (grey), E (light blue), and F (pink) in DBS samples.

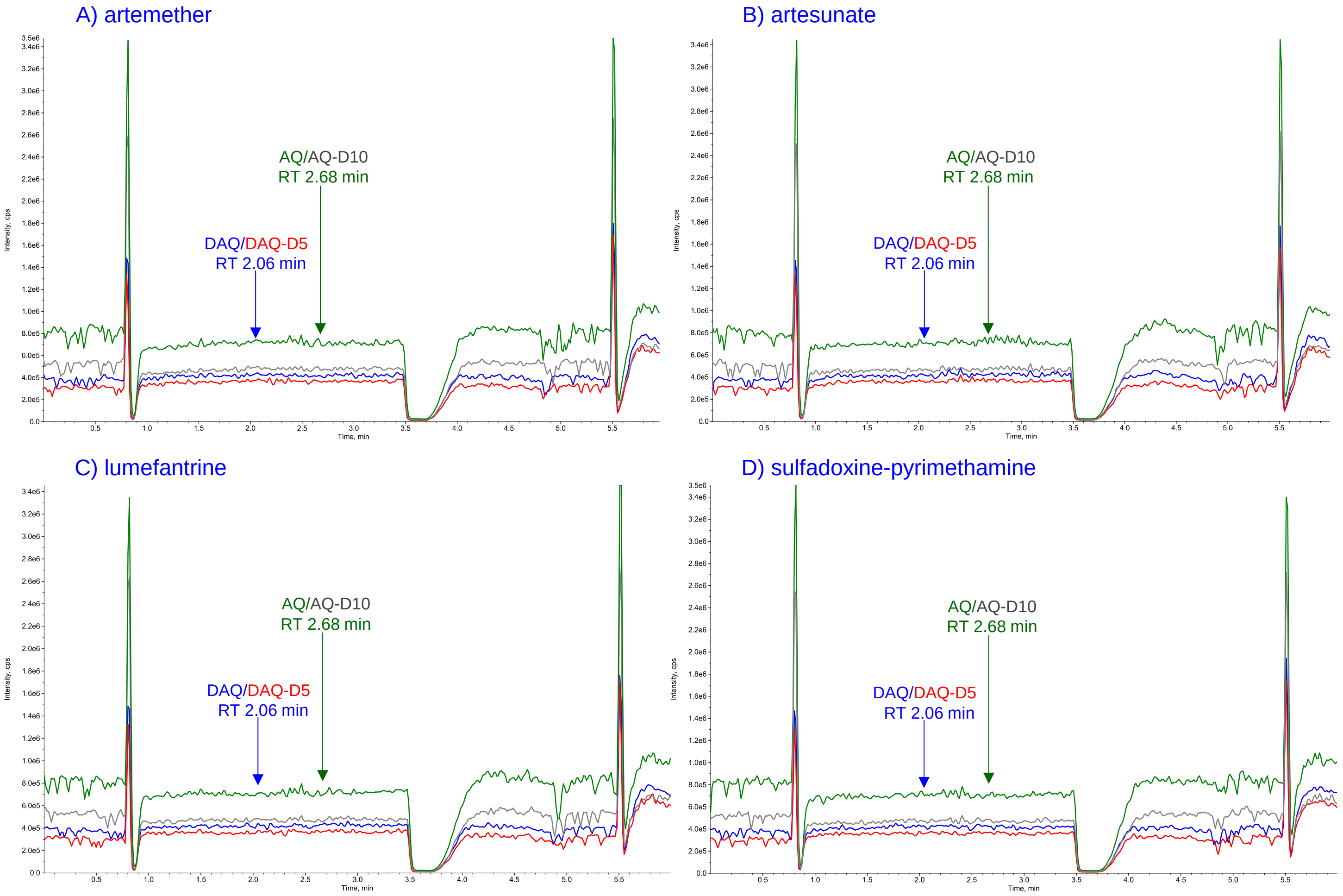

FIGURE S2: Extracted ion chromatogram of amodiaquine (AQ, green), desethylamodiaquine (DAQ, blue), and stable isotope-labelled internal standards; AQ-D10 (grey) and DAQ-D5 (red) of five possible co-administered drugs, injected as neat solutions during post-column infusion. A) artemether at 373 ng/ml, B) artesunate at 546 ng/ml, C) lumefantrine at 4,346 ng/ml, and D) sulfadoxine-pyrimethamine at 32,700 ng/ml. Co-administered drugs were injected during post-column infusion of amodiaquine (26.7 ng/ml), desethylamodiaquine (22.8 ng/ml), and AQ-D10 (20.6 ng/ml), DAQ-D5 (20.6 ng/ml).

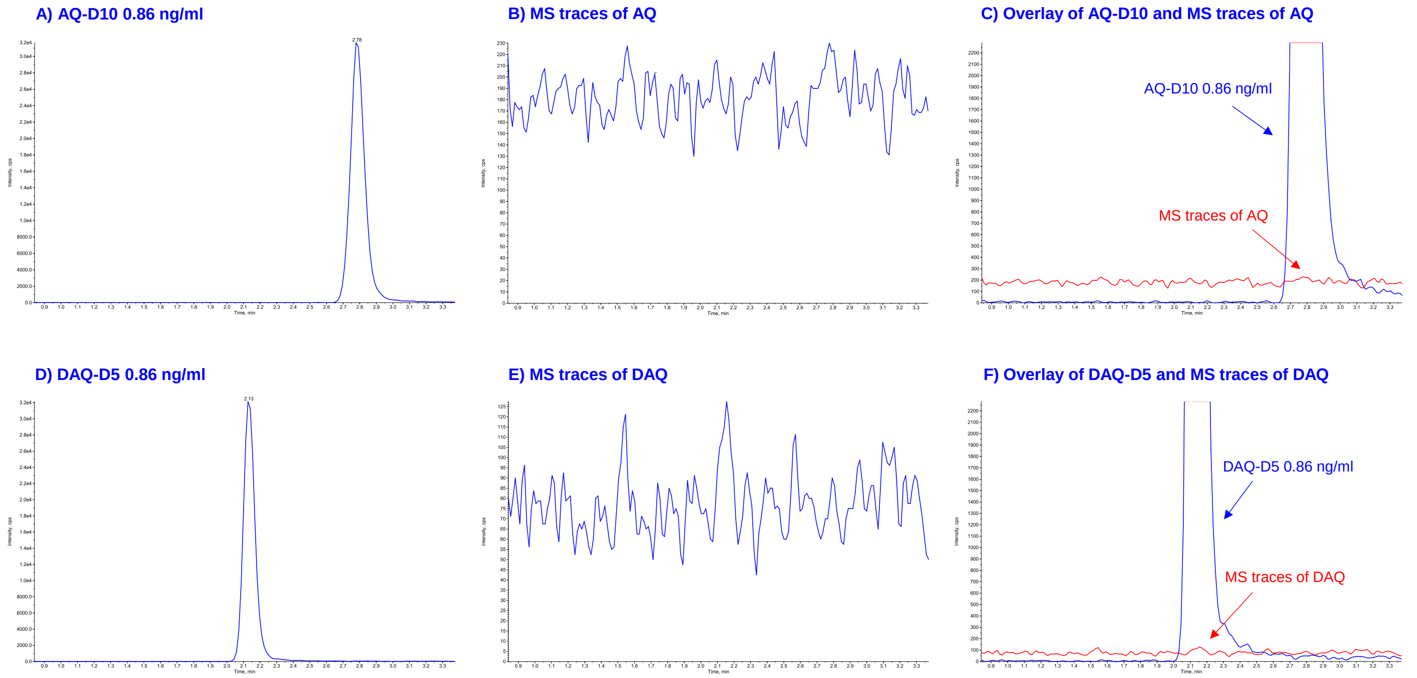

FIGURE S3: Extracted ion chromatograms for internal standards and analyte interference testing. The upper section shows A) AQ-D10 0.86 ng/ml, B) MS traces of AQ, and C) overlay of AQ-D10 at 0.86 ng/ml and MS traces of AQ. The lower section displays D) DAQ-D5 0.86 ng/ml, E) MS traces of DAQ, and F) Overlay of DAQ-D5 at 0.86 ng/ml and MS traces of DAQ. No interference was observed at the retention times of AQ and DAQ.

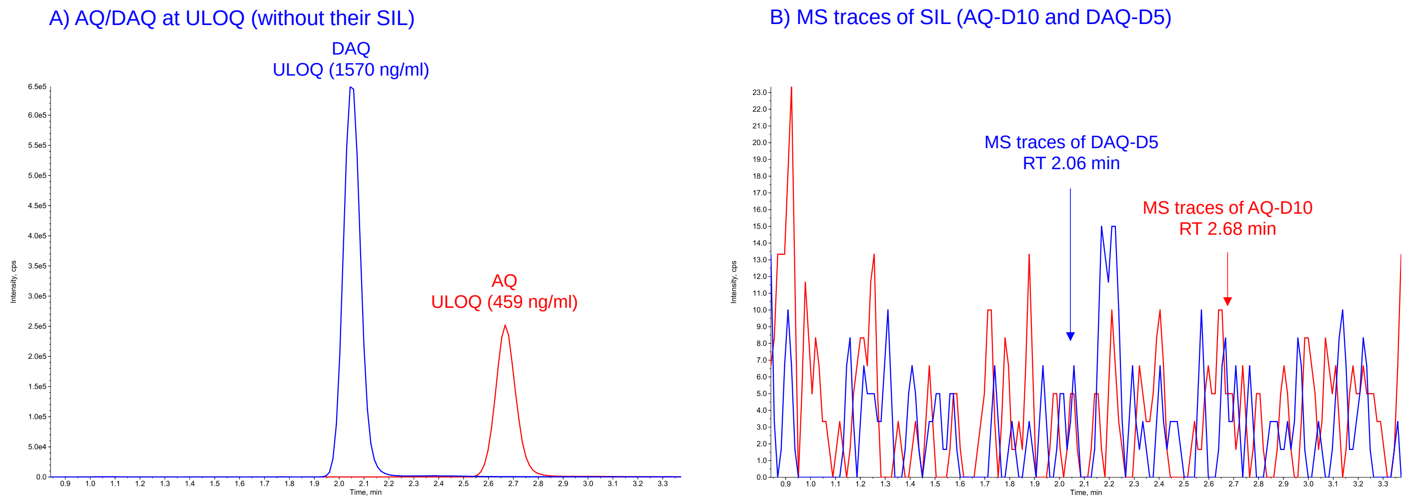

FIGURE S4: Extracted ion chromatograms for amodiaquine (AQ, red), desethylamodiaquine (DAQ, blue) interference testing. A) AQ/DAQ at ULOQ (without their SIL) in DBS sample and B) MS traces of SILs (AQ-D10 and DAQ-D5). No interference was detected from AQ and DAQ at ULOQ to their respective SILs.

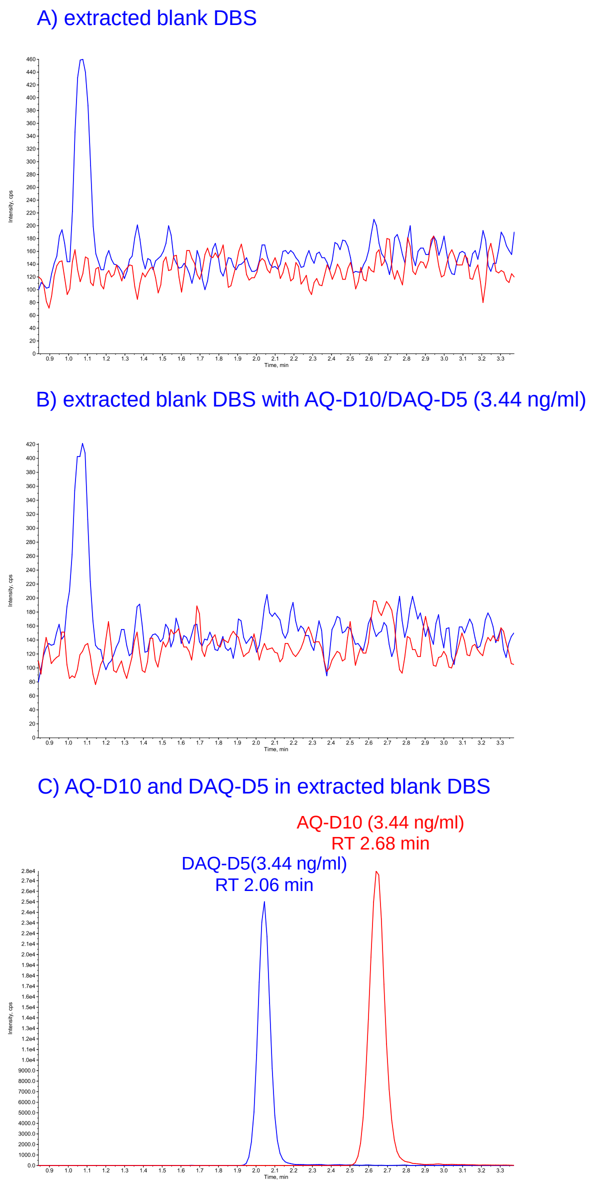

FIGURE S5: Extracted ion chromatograms of amodiaquine (AQ, red) and desethylamodiaquine (DAQ, blue) in blank DBS extraction with and without internal standards. A) extracted blank DBS (without internal standards), B) extracted blank DBS (with AQ-D10 and DAQ-D5 at 3.44 ng/ml) and C) AQ-D10 and DAQ-D5 (3.44 ng/ml) in extracted blank DBS with AQ-D10 and DAQ-D5 (3.44 ng/ml).
